# Understanding Lignin Dissolution with Urea and the Formation of a Lignin Nano-Aggregate: A Multiscale Approach

**DOI:** 10.3390/nano14070593

**Published:** 2024-03-27

**Authors:** Jinxin Lin, Liheng Chen, Yanlin Qin, Xueqing Qiu

**Affiliations:** 1 Guangdong Provincial Key Laboratory of Plant Resources Biorefinery, School of Chemical Engineering and Light Industry, Guangdong University of Technology, Guangzhou 510006, Chinaylqin@gdut.edu.cn (Y.Q.); 2 Guangdong Provincial Laboratory of Chemistry and Fine Chemical Engineering Jieyang Center, Jieyang 515200, China

**Keywords:** lignin nanoparticles, lignin conformation, lignin molecular dynamics, self-assembly of lignin

## Abstract

This study employs a combined computational and experimental approach to elucidate the mechanisms governing the interaction between lignin and urea, impacting lignin dissolution and subsequent aggregation behavior. Molecular dynamics (MD) simulations reveal how the urea concentration and temperature influence lignin conformation and interactions. Higher urea concentrations and temperatures promote lignin dispersion by disrupting intramolecular interactions and enhancing solvation. Density functional theory (DFT) calculations quantitatively assess the interaction energy between lignin and urea, supporting the findings from MD simulations. Anti-solvent precipitation demonstrates that increasing the urea concentration hinders the self-assembly of lignin nanoclusters. The findings provide valuable insights for optimizing lignin biorefinery processes by tailoring the urea concentration and temperature for efficient extraction and dispersion. Understanding the influence of urea on lignin behavior opens up avenues for designing novel lignin-based materials with tailored properties. This study highlights the potential for the synergetic application of MD simulations and DFT calculations to unravel complex material interactions at the atomic level.

## 1. Introduction

Lignin, the second most abundant biomacromolecule on Earth after cellulose, exhibits substantial potential as a renewable resource with diverse applications [1]. Nevertheless, the intricate structure and inherent recalcitrance of lignin present formidable challenges to its effective utilization [2]. Recent advancements in research have concentrated on elucidating the potential of lignin by scrutinizing its complex structure and behavior, specifically focusing on the formation and properties of nano-aggregates [3,4]. These nanoscale entities, arising from the self-assembly of lignin molecules, have garnered considerable attention due to their distinctive properties and potential applications across various domains [5,6]. In contrast to bulk lignin, nano-aggregates manifest unique characteristics attributable to their small size and large surface area, offering enhanced accessibility and functionalization potential compared to traditional lignin, which is often challenging to process due to its recalcitrance [7].

Researchers, through precise control of the aggregation process, can engineer lignin nanoparticles with tailored sizes and functionalities [6,8,9]. These engineered nanoparticles hold promise in diverse applications, spanning drug delivery, nanocomposites, and energy storage devices [10,11]. Notably, in drug delivery, lignin nanoparticles with controlled sizes and surface functionalities present significant potential; for instance, we may capitalize on their inherent biocompatibility and tunable properties for applications in targeted and controlled drug delivery, thereby minimizing potential side effects [10,12,13]. Moreover, lignin nano-aggregates, with their large surface areas and controlled functionalities, act as efficient reinforcing agents in composite materials, contributing to the development of sustainable and high-performance materials [14,15]. Their porous structures and surface functionalities also render them suitable for incorporation into energy storage devices like supercapacitors and batteries, offering avenues for enhancing the energy storage capacity and efficiency [16,17].

However, despite the promising applications of lignin nano-aggregates, the underlying mechanisms governing their formation remain elusive. This complexity arises from a multitude of factors, including the lignin sources [18], extraction methods [19,20], and aggregation conditions [21,22]. Parameters such as pH, temperature, ionic strength, and solvent molecular types significantly influence intermolecular interactions and self-assembly processes, ultimately impacting the size, shape, and morphology of the formed nano-aggregates [23,24,25]. Particularly noteworthy is the influence of the solvent used to dissolve lignin, as solvent molecules interact with lignin molecules, influencing their intermolecular interactions and self-assembly behavior [20,24,26]. The choice of solvent emerges as a critical factor for tailoring the properties of lignin aggregates for specific applications [23,24].

Urea, a readily available and cost-effective nitrogen fertilizer, has been demonstrated to facilitate lignin dissolution, presenting a promising avenue for lignin valorization and extraction from lignocellulosic biomasses [27]. The utilization of green solvents not only enhances the biocompatibility of lignin products, thereby extending their application potential, but also mitigates environmental harm throughout the production process [28]. Nonetheless, despite promising results in numerous studies, the intricate mechanisms underlying urea-induced lignin dissolution remain elusive. Researchers have employed various experimental techniques such as dynamic light scattering (DLS) and atomic force microscopy (AFM) to explore the mechanisms of lignin aggregation in urea solution [29]. Traditional experimental methods have provided valuable insights but often lacked the resolution to capture the dynamic and multifaceted nature of these interactions at the molecular level. Potentially overcoming this limitation, molecular dynamics (MD) simulations, offering unparalleled atomic-level detail, have emerged as a potent tool to bridge this gap [23,30,31,32].

In this study, we utilized MD simulations to track the formation and lifetime of hydrogen bonds between lignin and urea, providing crucial insights into the strength and specificity of their interactions. Additionally, we investigated changes in lignin conformation upon interaction with urea to unveil how urea disrupts inter-lignin interactions and promotes dispersion. Finally, MD simulations, in conjunction with density functional theory (DFT) calculations, were executed to determine the interaction energies between lignin and urea, revealing the thermodynamic driving forces behind their complexation. In providing these in-depth results, molecular simulations complement traditional experimental techniques, offering a deeper understanding of the mechanisms governing the interaction between lignin and urea. This knowledge is pivotal for optimizing pre-treatment processes in biorefineries and designing novel lignin-based nano-materials.

## 2. Materials and Methods

### 2.1. Materials

Corncob lignin was obtained as the residue of alkaline treatment at a biomass refinery of Shangdong Longlive Biotechnology Co., Ltd. (Dezhou, China) It molecular weight was about 6400 g/mol, as determined by gel permeation chromatography (GPC). More details of the corncob lignin were reported as alkaline lignin in our previous work [33]. Urea was purchased from Shanghai Maclin Biochemical Corporation (Shanghai, China). Ultra-pure water was employed throughout this study.

### 2.2. Methods

#### 2.2.1. Solubility Test of Corncob Lignin in Urea Aqueous Solution

Briefly, 0.5 g of corncob lignin was dissolved in a 50 wt% urea aqueous solution; then, this was transferred to a 500 mL volumetric flask and we added more 50 wt% urea solution to the graduation marking, to obtain 1 g/L of standard lignin urea aqueous water. The following lignin urea aqueous solutions with different lignin concentrations for the experiments were all diluted from this standard solution. The standard solution was firstly diluted into 0.001, 0.002, 0.005, 0.01, 0.015 and 0.02 g/L. Then, 20 mg/L of lignin standard solution was used to test for the absorbance from the 200 to 600 nm wavelengths via ultraviolet spectroscopy. The absorbance at the 278 nm wavelength of each lignin standard solution was collected, to obtain a standard curve of lignin solubility in the urea aqueous solution.

To determine the solubility of lignin in the urea solution, 5 mL of urea solution at a specific concentration (10, 20, 30, 40 and 50 wt%) was transferred into glass vials, which were then placed in a water bath set to the corresponding temperature. Upon reaching the desired temperature, an excess amount of lignin was added to the solution. The mixture was stirred using a magnetic stirrer for 1 h while keeping the vial sealed. After stirring, the supernatant was extracted using a syringe and rapidly filtered through an organic-based filter head to obtain the filtrate. Subsequently, the absorbance of the diluted filtrate was measured to calculate the solubility of lignin based on the standard curve.

#### 2.2.2. Equilibrium Simulation in Different Concentrations of Urea Solvents

**Figure 1:**
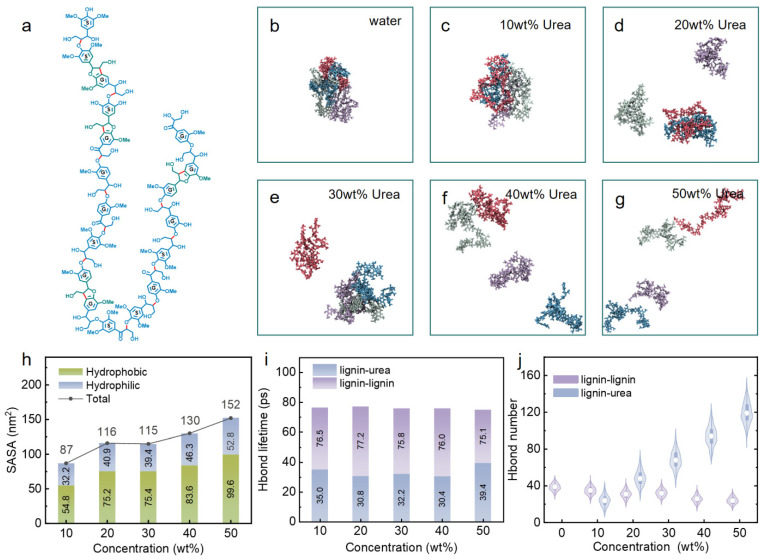
(**a**) Schematic 2D structure of linear corncob lignin model. Snapshots of 4 lignin molecules (each color represents a lignin molecule, Licorice model) dissolving in (**b**) water or (**c**) 10, (**d**) 20, (**e**) 30, (**f**) 40, or (**g**) 50 wt% urea solution at 353 K. (**h**) Solvent’s accessible surface area, (**i**) lifetime, and (**j**) number of hydrogen bonds.

Utilizing 2D HSQC NMR and gel permeation chromatography (GPC) data from previous reports [34], a linear lignin model (Figure 1a) with 18 units (including 7 syringyl and 11 guaiacyl units with 13 β-aryl ether and 4 phenylcounmaran linkages) was constructed using LigninBuilder [35] and CHARMM parameters [30,36]. Topology files for Gromacs were generated with TopoGromacs [37], and the CHARMM General Force Field (CGenFF) [38] described urea molecules.

The initial configuration of four lignin polymers was derived from the final simulation in water (Figure S1), simulated for 100 ns at constant temperature and pressure (298 K, 1 bar). To ensure the correct density was used, urea/water boxes with concentrations ranging from 10 to 50 wt% were prepared using the Packmol program and equilibrated in a constant-temperature and -pressure ensemble. Starting solvation boxes were constructed with the solvate command in GROMACS, placing initial lignin polymers at the center of 10 × 10 × 10 nm boxes.

Simulations were conducted using GROMACS v2023.01. Systems were energy-minimized, ensuring a maximum force of less than 50 kJ·mol^−1^·nm^−1^, and equilibrated at 298 K and 1 bar for 5 ns. Preceding 50 ns production simulations, 50 ns annealing runs in an isobaric–isothermal ensemble (NPT) were performed. The annealing temperature was set at 500 K. The LINCS algorithm constrained hydrogen bond lengths during production. A 12 Å cutoff was set for short-range electrostatics and van der Waals terms with a range 1.2 Å grid, with long-range electrostatic interactions treated by the particle mesh Ewald (PME). All simulations were maintained at a temperature of 298, 313, 333, 353, or 373 K using a V-rescale thermostat and Parrinello–Rahman barostat for pressure control at 1 bar. Periodic boundary conditions were applied.

#### 2.2.3. Analysis

GROMACS was used to analyze the solvent accessible surface area (SASA), hydrogen bond lifetime, hydrogen bond number, and interaction energy. Density contour images of the benzene ring–ring distance versus angle were sampled from the last 10 ns trajectories after the system equilibrium and interacting benzene rings within 5.5 Angstrom, and they were analyzed using the Python-enabled VMD v1.9.4a50 [39]. 

#### 2.2.4. Density Functional Theory (DFT) Calculations

To obtain the configurations with the lowest energy, thousands of configurations were sampled from a molecular dynamic run with GFN-FF via the extended tight binding (xTB) program package. The obtained clusters were then optimized with GFN0-xTB and we screened out hundreds of configurations using the Molclus program [40]. Furthermore, the clusters were reoptimized at the GFN2-xTB level with consideration of the water implicit solvent model. The configurations with a relative energy of within 3 kcal/mol were then optimized at B3LYP/6-31G* and confirmed without the imaginary frequency. Configurations with the lowest energy, used for quantum chemistry calculations, were optimized at the B97-3c [41] level using ORCA 4.2.1 software [42]. Interaction energies were calculated at B3LYP-D3/6-311+G** under the SMD water solvent model using Gaussian 16. To unveil weak interactions between guaiacyl-glycerol-beta-guaiacyl ether (GGE) and solvent molecules, non-covalent interaction (NCI) analyses [43], including the independent gradient model (IGM) [44] and reduced density gradient (RDG) method, were implemented through the Multiwfn 3.8(dev) [45] program and visualized via the Python-enabled VMD v1.9.4a50 [39].

#### 2.2.5. Preparation of Corncob Lignin Nanoparticles (CLNPs)

Corncob lignin nanoparticles were synthesized through a self-assembly technique in a solvent mixture of urea and water, wherein lignin dissolution occurred. Corncob lignin was dissolved in a urea/water solution (10, 20, 30, 40, 50 wt%) to create a lignin solution with the concentration set at 1 mg/mL. Subsequently, this solution was transferred to a dialysis bag featuring a molecular weight cut-off of 1000 Da. The bag was then immersed in ultra-pure water for 36 h, promoting the self-assembly of corncob lignin nanoparticles, denoted as CLNPs.

#### 2.2.6. Characterization of CLNPs

The hydrodynamic diameters of the CLNPs were analyzed using a Zetasizer NANO ZS particle analyzer. To observe the micromorphology of CLNPs, atomic force microscopy (AFM) with a Park NX10 instrument was employed.

## 3. Results and Discussion

### 3.1. Effect of Urea Content on Lignin Dissolution Behaviors in Urea Aqueous Solution

Alkaline lignin acquired in our investigation was dissolved in aqueous urea solutions with varying concentrations. As corroborated by the previous literature [29] and illustrated in Table 1, the solubility of lignin exhibited a gradual increment with the ascending urea concentration. Noteworthy is the observation that the alkaline lignin utilized in this study demonstrated near-insolubility in pure water. The pH values of urea aqueous solutions are presented in Table S1. Within the concentration range of 10–40 wt% urea, the pH gradually increased, but a slight decrease in pH was observed as the urea concentration reached 50 wt%. This decrease indicated a reduction in the concentration of free hydroxide ions, suggesting that at the 50 wt% urea concentration, a stronger hydrogen bonding network was formed between urea and water, thereby inhibiting the liberation of hydroxide ions. Generally, the pKa value of phenolic hydroxide is around 10. Hence, to facilitate the dissolution of alkaline lignin, a solution pH value of 10 or higher is typically required. Therefore, the enhanced solubility of lignin in urea solution can likely be attributed to the strong interaction between urea and lignin. Further analysis of the atomic-level interaction between urea molecules and lignin was warranted to elucidate the mechanism of lignin solvation by urea solution. In order to attain a molecular-level comprehension of the dissolution dynamics of alkaline lignin within this urea system, we initiated an exploration through molecular dynamics simulations. As illustrated in Figure 1b, in a pure water environment, lignin molecules engaged in interactions, leading to their aggregation into a pseudo-spherical structure. Upon raising the urea concentration from 10 wt% to 30 wt% in the solution, lignin molecules exhibited partial separation, yet the linear folding of the lignin model persisted (Figure 1c–e). At a urea concentration of 40 wt%, the lignin model molecules were nearly dispersed, but internal stacking remained evident (Figure 1f). With a further increase to a 50 wt% urea concentration, lignin molecules achieved complete dispersion, and while a few folded conformations persisted, the predominant conformation transitioned to a linear stretch (Figure 1g). The solvent-accessible surface area (SASA), a pivotal metric in the study of biomolecular folding and stability, was employed to scrutinize the impact of urea on lignin conformation. As shown in Figure 1h, the SASA of lignin generally increased with the rising urea concentration. The radius of gyration of lignin in the urea aqueous solution exhibited the same trend as the SASA (Figure S2). Simultaneously, at the same temperature, the lifetime of hydrogen bonds exhibited relative stability, unaffected by the presence of urea (Figure 1i). Calculating the number of hydrogen bonds formed between urea and lignin revealed a notable increase with the escalating urea concentration. In contrast, the count of hydrogen bonds between lignin molecules exhibited a slight decrease (Figure 1j). This observation suggests a positive correlation among changes in lignin conformation, solvation area, and hydrogen bond content.

**Figure 2:**
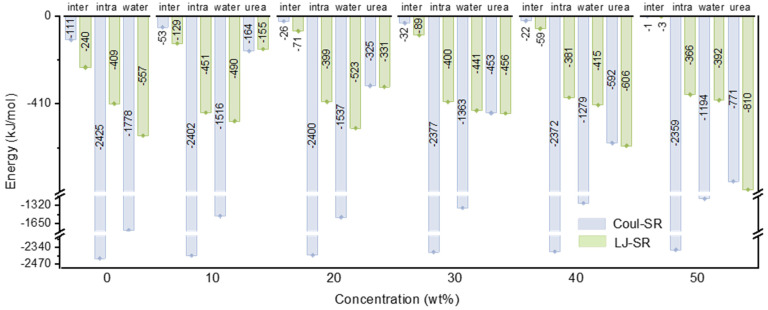
Inter-/intramolecular interaction of lignin dissolved in different solvent systems, and interaction energy between lignin and different solvent molecules.

From an energetic perspective (Figure 2), within the concentration range of 20–30 wt%, despite the increase in urea and lignin interaction from −656 kJ/mol (−325 kJ/mol Coulomb interaction and −331 kJ/mol van der Waals interaction) to −909 kJ/mol (−453 kJ/mol Coulomb interaction and −456 kJ/mol van der Waals interaction), both the intermolecular and intramolecular interactions of lignin essentially remained unaltered. Additionally, in this concentration variation, the interaction between lignin and water experienced a substantial decrease with the rise in urea concentration. Upon reaching a urea concentration of 40 wt%, both the intermolecular and intramolecular interactions of lignin witnessed significant reductions. It was noteworthy that the intermolecular interaction among lignin molecules primarily comprised van der Waals interactions (such as π–π stacking interactions within molecules), while the intramolecular interaction within lignin molecules predominantly consisted of electrostatic interactions, exemplified by hydrogen bonds within lignin molecules. The interaction between urea and lignin intensified with an increasing concentration. Interestingly, at a urea concentration of 10 wt%, although the discrepancy between Coulomb and LJ energy was not substantial, Coulomb interactions still predominated over LJ. However, with the continuous increase in the urea concentration, LJ gradually surpassed Coulomb, and the disparity continued to widen. Upon reaching a urea concentration of 50 wt%, the interaction energy between urea and lignin (−1581 kJ/mol) became comparable to that of water (−1586 kJ/mol), with the Coulomb term at −771 kJ/mol and the LJ term as high as −810 kJ/mol.

**Figure 3:**
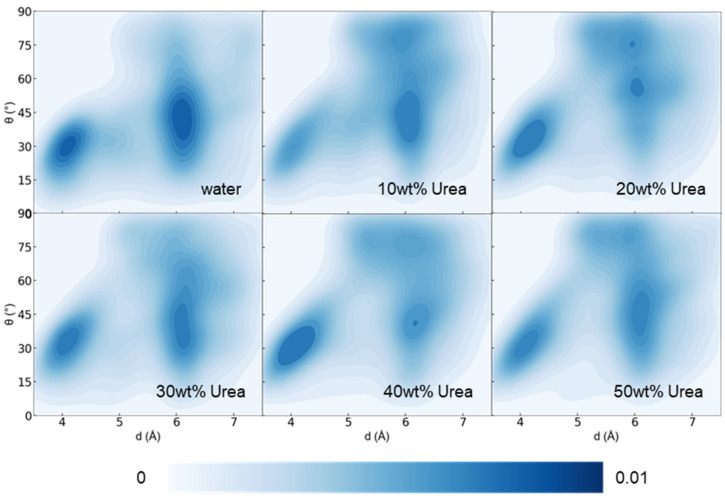
Density contour images of benzene ring–ring distance (between the mass center of all interacting (within 6 Angstrom) pairs of rings) versus benzene ring–ring stacking angle within lignin in different urea concentration systems at 353 K after 100 ns.

We further scrutinized the intramolecular interactions of lignin. Through an analysis of the normal vector and centroid distance of all interacting benzene rings (C atoms on the benzene ring in contact within 5 Å), as shown in Figure 3, despite a lightening of color in regions of less than 4 Å and 30°, the overall lower-left corner region remained conspicuous, affirming the persistence of intramolecular benzene ring interactions within lignin.

### 3.2. Effect of Temperature on Lignin Dissolution Behaviors in Urea Aqueous Solution

**Figure 4:**
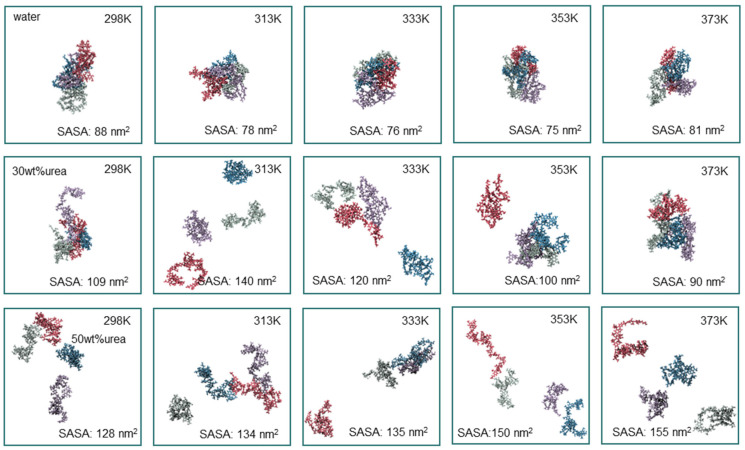
Snapshots after 100 ns of molecular dynamics simulation of four lignin molecule clusters dissolving in distinct temperatures and concentrations of urea aqueous solution (four colors represent four molecules of lignin model, Licorice model, with implicit urea aqueous solvent).

Temperature constitutes a crucial and easily controllable factor that significantly influences the solubility of lignin. As is evident in Table 2, the solubility of lignin gradually increased with rising temperatures in urea solutions. To delve deeper into the impact of temperature on lignin conformation, we conducted temperature gradient simulations under pure water and 30 wt% and 50 wt% urea solutions (Figure 4). In pure water and he 30 wt% urea solution, escalating temperatures led to the aggregation of lignin molecules, resulting in a decrease in the solvent-accessible surface area. However, at 313 K in the 30 wt% urea solution, lignin achieved complete dispersion, exhibiting the largest solvent-accessible surface area at this concentration. Further temperature increase caused the lignin in the 30 wt% concentration to gradually aggregate into a sphere-like structure. In contrast, at a 50 wt% urea concentration, a different trend emerged, with the solvent-accessible surface area continuously increasing with temperature, peaking at 155 nm^2^ at 373 K. The radius of gyration of lignin in the urea aqueous solution at 373 K followed a similar trend to that observed in SASA (Figure S3).

**Figure 5:**
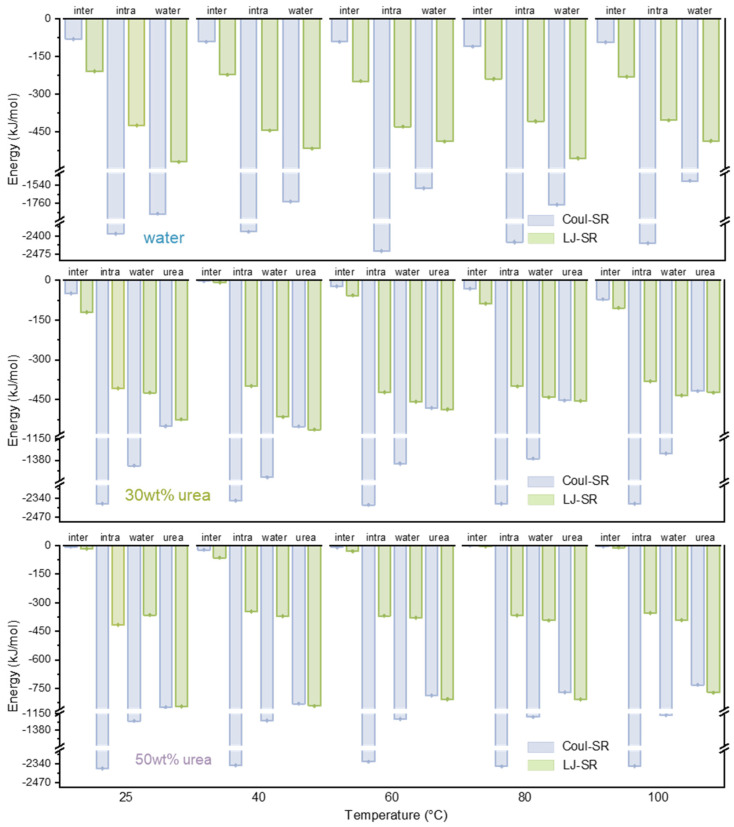
Inter-/intramolecular interaction of lignin dissolved in different solvent systems, and the interaction energy between lignin and different solvent molecules.

To understand the mechanisms behind these dynamics, we scrutinized the interaction energy (Figure 5). It was evident that the interactions between lignin molecules in water remained essentially unchanged. However, due to the intensified thermal motion and the influence of hydrophobic interactions with a rising temperature, more intramolecular hydrogen bonds formed within lignin molecules. The intramolecular van der Waals interaction remained relatively weak, without a significant increase. The thermal motion had the most pronounced effect on the interaction between lignin and urea. At a urea concentration of 30 wt%, gentle thermal motion at 40 °C facilitated a more comprehensive interaction between lignin and solvent molecules. The interaction energy between lignin, urea and water molecules significantly increased, particularly the van der Waals interaction between lignin and urea, aiding the dispersion of lignin molecules. As the temperature increased to 40 °C, the interaction between urea and lignin notably decreased, attributable to the instability of hydrogen bonds at higher temperatures. At a urea concentration of 50 wt%, the abundance of urea molecules compensated for the low lifetime of hydrogen bonds at high temperatures. At this point, the interaction between urea and lignin remained relatively stable, with the van der Waals interaction dominating over the electrostatic interaction, contributing to maintaining the stretched conformation of lignin during thermal motion.

**Figure 6:**
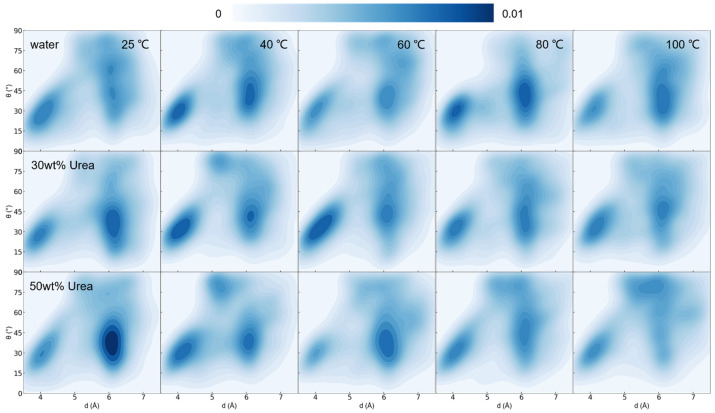
Density contour images of benzene ring–ring distance (between the mass centers of all interacting (within 6 Angstrom) pairs of rings) versus benzene ring–ring stacking angle within lignin in water, for the 30 and 50 wt% urea concentration systems at 25, 40, 60, 80, and 100 °C after 100 ns.

In the analysis of benzene ring interactions (Figure 6), the distribution density in pure water remained largely unchanged from 25 to 100 °C. At a urea concentration of 30 wt%, although the distribution exhibited an upward shift at 40 °C, the overall π–π interaction was sustained. Conversely, at a concentration of 50 wt%, while the π–π interaction persisted, the dark area displayed a general upward trend, indicating a significant weakening of interactions between benzene rings.

### 3.3. Effect of Urea on Lignin Self-Assembly by an Anti-Solvent

**Figure 7:**
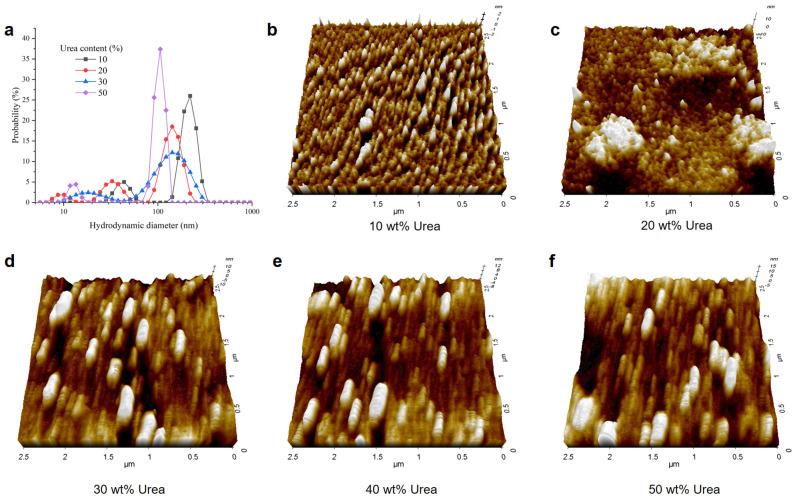
(**a**) Hydrodynamic diameter of lignin nano-aggregate dispersed in water from the lignin–urea aqueous solution. (**b**–**f**) AFM images of CLNPs.

Through the preceding molecular dynamics investigations, it was evident that the conformation of lignin varied in different urea solutions. Understanding the impact of these variations on the subsequent preparation of lignin nanoparticles through an anti-solvent method is pivotal, as it can guide the regulation of lignin nanoclusters. Consequently, we subjected lignin dissolved in urea solutions of different concentrations to a self-assembly process through dialysis in water to study its aggregation behavior. Figure 7a depicts the hydrodynamic diameter of lignin water dispersions obtained through the anti-solvent method, with particle sizes predominantly distributed within 500 nm and zeta potentials of about −30 mV. The AFM images in Figure 7b–f reveal that the obtained lignin nano-aggregate was not perfectly regular. Overall, as the urea concentration for dissolving lignin increased, the particles obtained through the anti-solvent method gradually decreased in size. Interestingly, with the rising urea concentration for dissolving lignin, it became challenging to precipitate the obtained lignin water dispersions through centrifugation. This suggested that, with the increase in urea content, the interaction between lignin and urea molecules intensifies, manifested by an enhanced solvation effect. This observation aligned with the conclusions drawn from the earlier dynamic simulation study. To quantitatively explore such solvation effects in the future, more sophisticated methods will be required to delve into their direct interactions.

### 3.4. Non-Covalent Interactions (NCIs) between Urea and Lignin Unveiled by DFT

**Figure 8:**
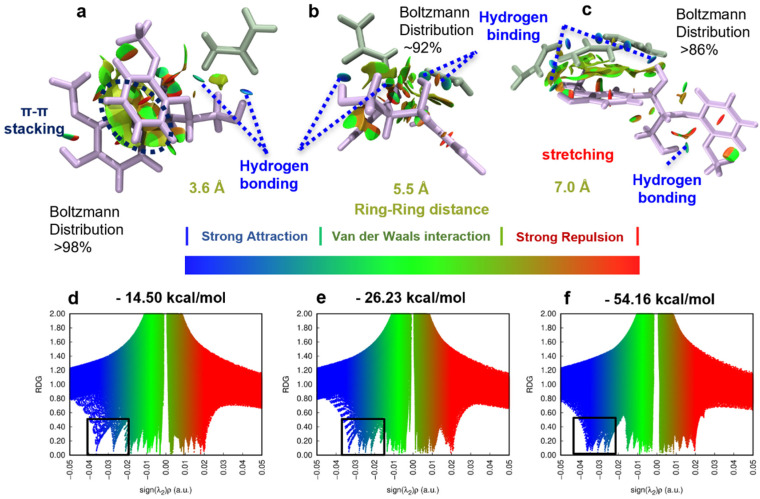
Theoretical configurations of the lignin dimer model with (**a**,**d**) 1, (**b**,**e**) 2, and (**c**,**f**) 3 urea molecules representing distinct concentration solvent systems. (**a**–**c**) Iso-surface (value = 0.5) and (**d**–**f**) scatter graph of reduced density gradient (RDG) of GGE configurations in the implicit water solvent (GGE: Licorice model, purple; Urea: Licorice model, green; black box region: hydrogen bonding).

To achieve a more precise quantification of the interaction energy between lignin and urea molecules and to gain a deeper understanding of the mechanism governing the impact of the urea concentration on lignin dissolution, we employed density functional theory (DFT) for computational analysis. Given that G units and β-O-4 linkages are predominant in the selected corncob lignin, guaiacyl-glycerol-beta-guaiacyl ether (GGE) was selected as the lignin dimer model (Figure 8). Utilizing water as the implicit solvent model, we determined the lowest energy configurations of the lignin dimer model GGE when interacting with 1, 2, and 3 urea molecules, representing different concentrations of urea in the solvent environment. The reduced-density gradient (RDG) model was employed to study the non-covalent interactions (NCIs) both between and within lignin molecules.

In Figure 8a, the large green iso-surface indicates a robust van der Waals interaction (π–π stacking) between benzene rings, as well as van der Waals interactions between the substituents on benzene rings and H atoms on benzene rings. With a single urea molecule (Figure 8a), hydrogen bonds formed between urea and the O_β_ and O_γ_-H of GGE through its N-H and O, but these were insufficient to disrupt the interactions between the benzene rings of the lignin dimer. With an increase to two urea molecules (Figure 8b), robust hydrogen bonds formed between urea and the O_α_, O_β_, and O_γ_ of lignin. Benzene rings of the lignin dimer stretched apart, forming intramolecular hydrogen bonds between O_γ_-H and O on the benzene rings, along with hydrogen bonds between O_α_-H of urea molecules and lignin. Simultaneously, strong van der Waals interactions occurred between the planes of urea molecules and the benzene rings of lignin. As the urea molecules were increased to three, they formed a “large plane” primarily through N-H…O hydrogen bonds, displaying a substantial C-π van der Waals interaction area with the benzene rings of lignin. At this juncture, the inter-benzene ring distance of the lignin dimer increased from 3.6 Å to 7.0 Å, accompanied by a rise in the number of hydrogen bonds, evident in the increase in the number of blue peaks in Figure 8d–f. The interaction energy between urea and the dimer increased from −14.50 kcal/mol to −54.16 kcal/mol. The stacking and stretching conformations of lignin were intricately linked not only to the number and strength of hydrogen bonds formed but also to the van der Waals interactions provided by the solution. As the urea concentration increased, the van der Waals interactions within the lignin molecules were disrupted by the synergistic effect of hydrogen bonds and van der Waals interactions of urea molecules, resulting in a stretched conformation.

**Figure 9:**
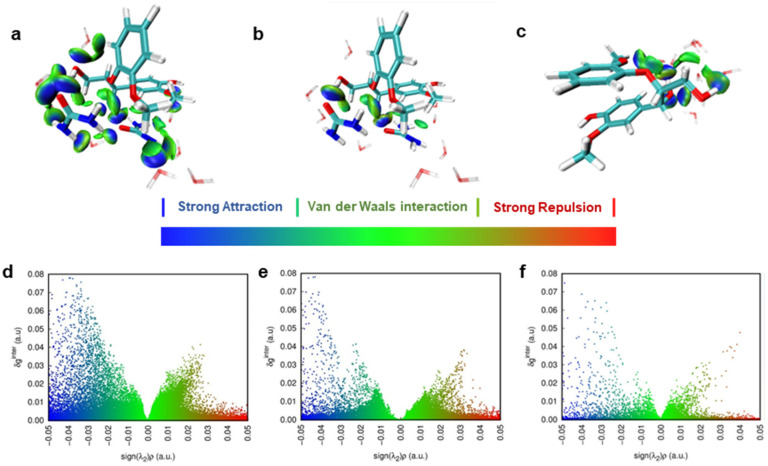
(**a**–**c**) Iso-surface (value = 0.05) and (**d**–**f**) scatter graph of independent gradient model (IGM) based non-covalent interaction between GGE and simulated urea aqueous solution (including 2 urea and 10 water molecules). Interactions between (**a**) GGE and solution, (**b**) GGE and urea molecules, and (**c**) GGE and only water molecules (Licorice model, C—cyan, H—white, O—red, N—blue).

We extended our simulations to encompass an explicit solvent environment (Figure 9). Compared with the lowest energy configuration under pure water, hydrogen bonds formed significantly between the O of urea molecules and formed O_γ_-H between GGE molecules, N-H between urea molecules, and between the O_β_ for GGE molecules. These interactions induced the opening of lignin benzene rings. Through molecular simulation research, we unraveled the mechanism by which the urea concentration influences lignin dissolution behavior. Urea disrupted the van der Waals interactions between lignin molecules and facilitated the formation of hydrogen bonds between them, leading to the stretching and dispersion of lignin conformation. The substantial interaction energy between urea and lignin not only induced lignin dissolution but also hindered the self-assembly of lignin, making it challenging to separate from water. This suggests that the regulation of lignin aggregation in water can be achieved by adjusting the concentration of dissolved urea.

## 4. Conclusions

In conclusion, this study sheds light on the complex interactions between lignin and urea, a promising solvent for lignin valorization. By combining molecular dynamics (MD) simulations, density functional theory (DFT) calculations, and experimental analysis, we have revealed critical factors influencing lignin dissolution and subsequent self-assembly behavior. MD simulations have demonstrated that the urea concentration and temperature significantly impact the lignin conformation and interactions. Higher urea concentrations and temperatures promote lignin dispersion by disrupting intramolecular interactions and enhancing solvation. DFT calculations supported these observations, quantifying the strengthening interaction energy between lignin and urea with an increasing concentration. Experimentally, increasing the urea concentration for initial lignin dissolution hindered the formation of lignin nanoclusters during anti-solvent precipitation. This study highlights the power of combining MD simulations and DFT calculations to unravel complex material interactions at the atomic level. Future research efforts should explore the influence of other solvent systems and delve deeper into the dynamics of lignin self-assembly, paving the way for further advancements in sustainable bio-based nano-material development.

## Figures and Tables

**Table 1:** Lignin solubilities in urea aqueous solutions with different concentrations at 333 K.

Urea Aqueous Solution Concentration (wt%)	10	20	30	40	50
Lignin solubility (mg/mL)	0.87	2.49	2.99	3.23	5.68

**Table 2:** Lignin solubilities in different temperatures of 50 wt% urea aqueous solutions.

Solution Temperature (K)	298	313	333	353
Lignin solubility (mg/mL)	1.34	3.56	5.68	12.13

## Data Availability

Data is contained within the article or Supplementary Material.

## Supplementary Materials

The following supporting information can be downloaded at: https://www.mdpi.com/article/10.3390/nano14070593/s1, Figure S1. Example of the initial structure of 4 lignin molecules in a urea aqueous solvent. Figure S2. Time-dependent radius of gyration of lignin in urea aqueous solution with different urea concentration at 353 K. Figure S3. Time-dependent radius of gyration of lignin in urea aqueous solution at different temperature. Table S1. pH value in urea aqueous solutions with different concentration. Table S2. Zeta potentials of lignin nano-aggregates produced from urea aqueous solutions with different concentration.
