# Supplementary material for: Understanding Lignin Dissolution with Urea and the Formation of a Lignin Nano-Aggregate: A Multiscale Approach
Source: Nanomaterials (Basel). 2024 Mar 27;14(7):593. doi: 10.3390/nano14070593 (PMC11013285; doi:10.3390/nano14070593)
Supplement: Supplementary file 1 [file nanomaterials-14-00593-s001.zip › nanomaterials-2925151-supplementary.pdf]

nanomaterials

Supplementary Materials

Understanding Lignin Dissolution with Urea and the Formation of a Lignin Nano-Aggregate: A Multiscale Approach

Jinxin Lin [1], Liheng Chen [1,2,*], Yanlin Qin [1,2] and Xueqing Qiu [1,2,*]

[1] Guangdong Provincial Key Laboratory of Plant Resources Biorefinery, School of Chemical Engineering and Light Industry, Guangdong University of Technology, Guangzhou 510006, China; ylqin@gdut.edu.cn (Y.Q.)
[2] Guangdong Provincial Laboratory of Chemistry and Fine Chemical Engineering Jieyang Center, Jieyang 515200, China
* Correspondence: lihengchen@gdut.edu.cn (L.C.); cexqqiu@scut.edu.cn (X.Q.)

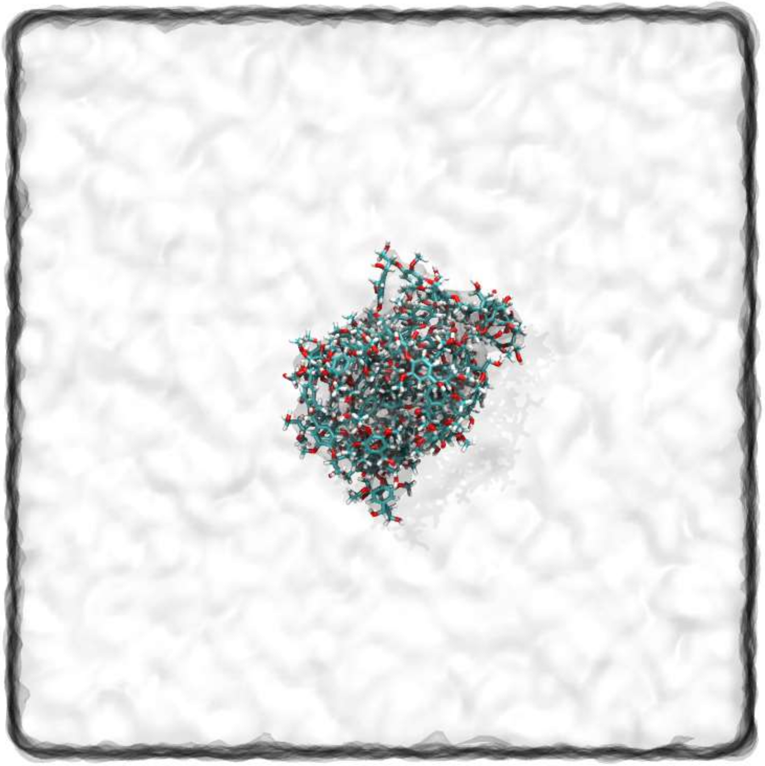

Figure S1. Example of the initial structure of 4 lignin molecules in a urea aqueous solvent.

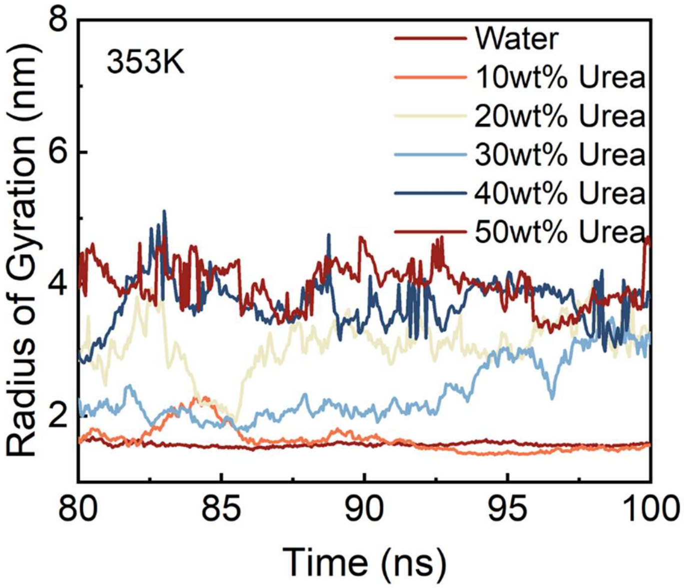

Figure S2. Time-dependent radius of gyration of lignin in urea aqueous solution with different urea concentration at 353K.

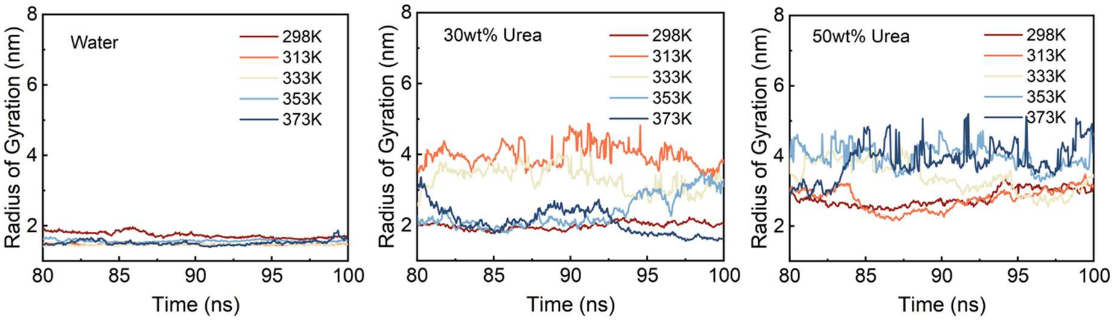

Figure S3. Time-dependent radius of gyration of lignin in urea aqueous solution at different temperature.

Table S1. pH value in urea aqueous solutions with different concentration.

Urea aqueous solution concentration (wt%)	10	20	30	40	50
pH	7.18	7.54	8.08	8.33	8.19

Table S2. Zeta potentials of lignin nano-aggregates produced from urea aqueous solutions with different concentration.

Urea aqueous solution concentration (wt%)	10	20	30	40
Zeta potential (mV)	–33.2±0.9	–32.1±0.2	–26.4±1.4	–34.9±1.4
